# Measurement and Analysis of Gait Pattern during Stair Walk for Improvement of Robotic Locomotion Rehabilitation System

**DOI:** 10.1155/2019/1495289

**Published:** 2019-10-14

**Authors:** Sang-Eun Park, Ye-Ji Ho, Min Ho Chun, Jaesoon Choi, Youngjin Moon

**Affiliations:** ^1^Biomedical Engineering Research Center, Asan Institute for Life Sciences, Asan Medical Center, Seoul, Republic of Korea; ^2^Department of Rehabilitation Medicine, Asan Medical Center, University of Ulsan College of Medicine, Seoul, Republic of Korea; ^3^Department of Biomedical Engineering, College of Medicine, University of Ulsan, Seoul, Republic of Korea; ^4^Department of Convergence Medicine, College of Medicine, University of Ulsan, Seoul, Republic of Korea

## Abstract

**Background:**

Robotic locomotion rehabilitation systems have been used for gait training in patients who have had a stroke. Most commercialized systems allow patients to perform simple exercises such as balancing or level walking, but an additional function such as stair-walk training is required to provide a wide range of recovery cycle rehabilitation. In this study, we analyzed stair-gait patterns and applied the result to a robotic rehabilitation system that can provide a vertical motion of footplates.

**Methods:**

To obtain applicable data for the robotic system with vertically movable footplates, stair-walk action was measured using an optical marker-based motion capture system. The spatial position data of joints during stair walking was obtained from six healthy adults who participated in the experiment. The measured marker data were converted into joint kinematic data by using an algorithm that included resampling and normalization. The spatial position data are represented as angular trajectories and the relative displacement of each joint on the anatomical sagittal plane and movements of hip joints on the anatomical transverse plane.

**Results:**

The average range of motion (ROM) of each joint was estimated as (−6.75°, 48.69°) at the hip, (8.20°, 93.78°) at the knee, and (−17.78°, 11.75°) at the ankle during ascent and as (6.41°, 31.67°) at the hip, (7.38°, 91.93°) at the knee, and (−24.89°, 24.18°) at the ankle during descent. Additionally, we attempted to create a more natural stair-gait pattern by analyzing the movement of the hip on the anatomical transverse plane. The hip movements were estimated to within ±1.57 cm and ±2.00 cm for hip translation and to within ±2.52° and ±2.70° for hip rotation during stair ascent and stair descent, respectively.

**Conclusions:**

Based on the results, standard patterns of stair ascent and stair descent were derived and applied to a lower-limb rehabilitation robot with vertically movable footplates. The relative trajectory from the experiment ascertained that the function of stair walking in the robotic system properly worked within a normal ROM.

## 1. Background

According to a report by the United Nations, every year, more than 795,000 people in the United States have a stroke. Stroke patients 85 years of age and older make up 17% of all stroke patients. The worldwide percentage of the population 65 years of age or older is projected to grow from 9.1% to 15.9% between 2015 and 2050. Because of rapid aging, over the period from 2010 to 2050, the number of incident strokes is expected to more than double [1, 2]. Strokes are the most representative cause of serious long-term disabilities such as hemiplegia in adults. Therefore, rehabilitation of locomotion is one of the main goals for people who have had a stroke. Traditional therapies usually focus on treadmill training to restore the functional mobility of the affected limbs [3, 4]. During such rehabilitation training, a patient is made to stand on a treadmill with his/her body supported by a suspension system [5], and several physiotherapists make and/or assist the walking movements of the patients' legs by manual handwork [6, 7]. However, the task is very difficult and laborious for therapists, and the procedure is complex to the extent that their excessive burden can lead to inconsistent quality of the task or reduced duration of net training. For these reasons, various robotic locomotion therapy systems have been developed, and some of them have been used to train patients in the clinical field [8–11].

Usually, these systems are based on treadmill-type trainers in combination with exoskeletons and body weight support (BWS) systems. The Lokomat® (Hocoma AG, Switzerland) uses linear actuators that control the joint angles at the hip and knee. The system is synchronized with the speed of the treadmill to assure precise matching between the speed of the orthosis and the treadmill [12–14]. Similarly, the ReoAmbulator™ (Motorika, USA) employs powered leg orthosis and robotic arms, which enable patients to contribute during walking on the treadmill. The robotic arms are attached laterally to the thigh and shank of the patient for control of the lower limbs [15, 16]. The LokoHelp (Lokohelp Group, Germany) aids the gait-training program on the treadmill without the use of exoskeletons on a patients' legs. It consists of an ankle orthosis for foot-drop prevention and a harness [17]. Such treadmill-type devices provide training programs exclusively for level walking owing to their mechanical structure.

In traditional rehabilitation, therapists allow patients to perform special gaits such as ascending or descending stairs. This training is more effective in improving the gait ability of patients with low severity impairments than simple exercises or level walking because the activities require more muscle strength, balancing abilities, and complex movements [9, 18–20]. However, such an additional function can be aided by just a few robotic systems of the footplate type. The G-EO System™ (Reha Technology AG, Switzerland) is composed of robotic end-effector devices that allow simulation of stair ascent and stair descent with a BWS system [21]. The GaitMaster5 system by the University of Tsukuba in Japan, is a lower-limb orthosis system; the patient straps his/her feet into pads connected to motion platforms. These platforms can move the user's foot forward (simulating walking) or up and down, similar to climbing [22]. The footplates guide the feet, thereby reproducing the gait trajectory of the ankle joint. These technologies tend to focus on movements of the ankle joint; furthermore, the absence of an exoskeleton or other structure that can control the hip and knee does not allow support of the joints. As a result, it may become challenging for patients to train correctly and effectively using systems where those joints are unconstrained [10].

The robotic lower-limb rehabilitation system gait trainer, M181-1, was developed by Cyborg-Lab, Korea [23]. The system facilitates level walking using robotic linkages and separate left and right footplates that track a patient's foot motion on the ground plane. As an improvement in the functionality of the system, the function of stair walking can be considered and a rehabilitation system that includes stair walking is expected to actively train patients. This rehabilitation system is a hybrid of the footplate and treadmill types because the system has footplates but the feet of a user do not always touch the plates. If the footplates of the robot are vertically and independently controlled, the patient can train not only for level walking but also for stair walking. In other words, this robotic system can be designed to provide patients with various gait exercises by combining exoskeletal links with spatially movable footplates.

In this study, a standard gait pattern of stair walking was created and converted into applicable data that implemented the stair-walking function in the M181-1 system. Thus, this study focused on the analysis of joint movement in stair ascent and stair descent for the application to the joint actuators of the robotic locomotion rehabilitation system. The first step of the protocol involved an experiment to acquire motion data using a motion capture system. The second was processing the data and calculating the parameters on the anatomical sagittal and transverse planes. Finally, the average of each motion parameter was estimated as a standard stair-walk pattern.

## 2. Methods

**Figure 1:**
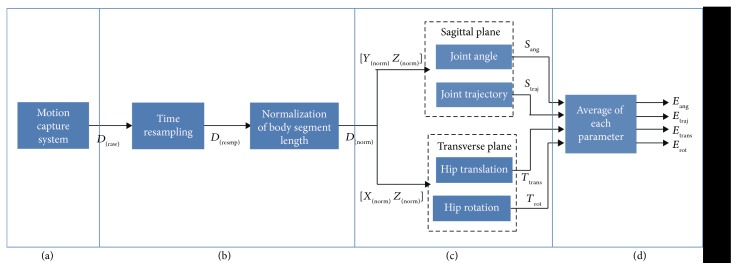
Protocol for analyzing a stair-walk pattern: (a) experiment and data acquisition with a motion capture system, (b) normalization of time and body segment length, (c) calculation of each parameter to analyze motion during stair ascent/descent, and (d) averaging every dataset to unify stair-gait pattern.

To make a patient train with a natural gait pattern, hip motion in the medial-lateral direction and hip rotation, as well as the movement of each joint on the sagittal plane, need to be applied to the robot.  Figure 1 indicates the process of analyzing stair-gait motion. The protocol has four steps: (a) position data acquisition, (b) data rescaling on the time and body segment length, (c) calculation of parameters for motion analysis, and (d) creation of a standard gait pattern.

### 2.1. Experiment for Data Acquisition

**Figure 2:**
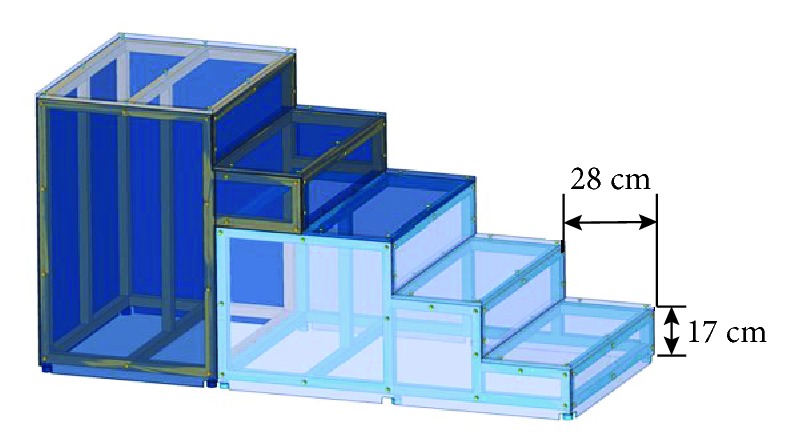
The experimental staircase was designed to have five steps. It had a 17 cm riser height and a 28 cm tread length according to the Korean building standards law.

For the test, a laboratory staircase composed of five steps and having a riser height and tread length of 17 cm and 28 cm, respectively, was prepared according to the Korean building standards law [24]. The prepared staircase is shown in Figure 2. Six healthy participants, four males and two females, participated in this study.  Table 1 summarizes information about the subjects.

To generate a reference standard gait pattern, the experiment was planned with subjects having no disorders in their lower limbs. The subjects were asked to repeatedly ascend and descend stairs at a self-selected velocity (normal pace) five times. The mean stride speeds were approximately 0.88 m/s in stair ascent and 0.96 m/s in stair descent. The method of stair walking was step-to-step, and a stride cycle was defined as the motion from the contact of the right foot of the first (third) step to the foot contact of the third (fifth) step, as described in [25]. Briefly, two cycles of stair-gaits were measured from the six subjects.

The highly complicated structure of the human skeleton enables movement with high degrees of freedom. Each body part moves in an unpredictable and complex motion trajectory. There are many types of systems for measuring body movements, such as optical marker-based tracking systems, markerless visual systems, and inertial measurement unit- (IMU-) based systems, which can be used to capture irregular human motion [26]. Because the optical marker-based system is frequently used in medicine [27–29] owing to its relatively high accuracy and minimal uncertainty of the subject's movement, the optical marker-based system was used to measure the normal stair-gait pattern in this study.

**Figure 3:**
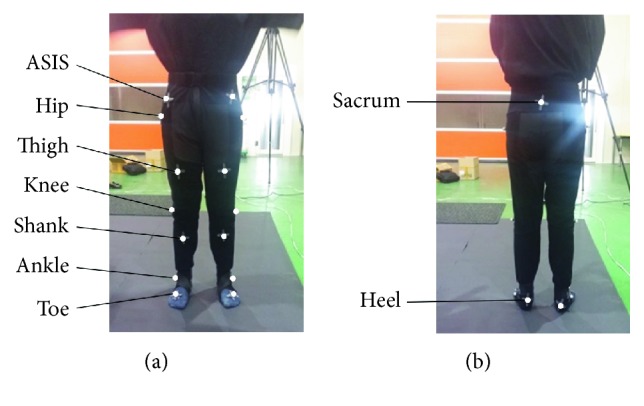
Markers were placed on a subject at the hip, thigh, knee, shank, ankle, and toe on both the right and the left sides including ASIS. (a) Front side. (b) Back side.

To acquire the position data of each joint in three-dimensional (3D) space, 17 optical markers were placed, one on the subject's sacrum, and two on the left and right anterior superior iliac spine (ASIS), hip, thigh, knee, shank, ankle, heel, and toe.  Figure 3 presents the arrangement of the markers on the front and back sides of a subject. The placements of the reflective markers were determined for accurate tracking of anatomical landmarks related to kinematic variables during gait [31–34].

**Figure 4:**
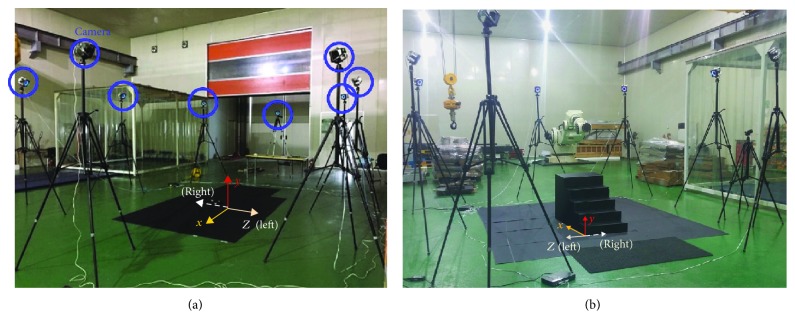
(a) Experimental environment for a camera setup (blue circles). (b) Position of the staircase. Yellow, red, and white arrows on the figures define the axes in coordinate space.

During the experiment, the positional information of the markers on the subjects was recorded at a rate of 160 Hz using a Prime 41 (OptiTrack, NaturalPoint Inc., USA) 3D motion capture system. The accuracy of this equipment is submillimeter, with a latency of 5.5 ms [30]. The calibration was performed with errors less than 2 mm. As shown in Figure 4(a), eight cameras, marked in blue circles, were placed in a square with approximate dimensions of 10 m × 10 m. The *x*-axis was defined as the direction of walking, with the *y*-axis as the vertical direction. The direction of the right (negative value) and left sides (positive value) was defined as the *z*-axis. The experimental staircase was installed at the center of the square.

The datasets *D*_(raw)_ = [*X*_(raw)_*Y*_(raw)_*Z*_(raw)_] measured by the motion capture system consisted of the *x*, *y*, and *z* coordinates for one cycle of stair walking. Each portion of the datasets, *X*_(raw)_, *Y*_(raw)_, and *Z*_(raw)_, denoted by time-series data for the attached 17 markers, was expressed by *X*_(raw)_ ∈ *R*^17×*N*^, *Y*_(raw)_ ∈ *R*^17×*N*^, and *Z*_(raw)_ ∈ *R*^17×*N*^, where *N* is the number of data points recorded for each marker. The value of *N* was different among the obtained datasets because of each participant's walking speed. In this study, the datasets were obtained for the six subjects who completed two stride cycles of stair ascent and descent a total of five times. Thus, a total 60 datasets of *D*_(raw)_ (6 subjects × 5 times × 2 cycles = 60 sets) were used for motion analysis of stair ascent and stair descent.

### 2.2. Data Preprocessing for Normalization

Because of the participants' own habits in walking, the walking velocity varied per person or trial. The lengths of body segments and the gap between the joints were also different among the participants. Therefore, it was necessary to normalize the data for time and space to simplify various conditions.

**Figure 5:**
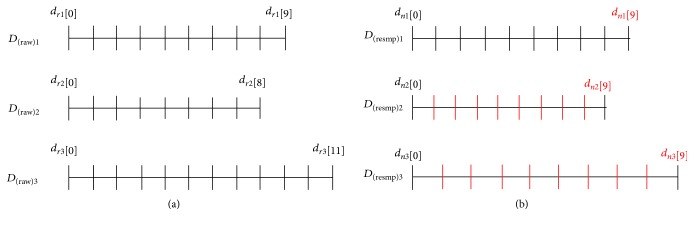
(a) Example of resampling datasets that have different lengths. (b) The vertical red lines are replaced using points by the cubic spline algorithm.

To unify the stride time condition, every *D*_(raw)_ was resampled to dataset *D*_(resmp)_ = [*X*_(resmp)_ *Y*_(resmp)_ *Z*_(resmp)_] with the same number (*M*) of components by applying the interpolation method of a cubic spline. The cubic spline is a function constructed of piecewise third-order polynomials that are smoother and have smaller errors than some other interpolating polynomials [35, 36].  Figure 5 shows an example of resampling the data *D*_(raw)*k*_[*m*] (*k* = 1, 2, and 3 and *m* = 0, 1, ⋯, *M*_*k*_ − 1, where *k* and *M*_*k*_ are constants), which is measured with the same sampling frequency but with a different length *M*_*k*_. *D*_(resmp)*k*_ is a modified dataset with the same number of samples (*M* = 10 in the example). To analyze the gait motion, the duration of a stride was divided into several sequences by physical and functional properties, such as period, i.e., stance and swing. The temporal unit was Stride cycle (%) for the analysis [20, 33, 37]. Therefore, the components of *D*_(resmp)_[*m*] (*m* = 0, ⋯, *M* − 1, where *M* is a constant) are considered as the identical functional sequence of gait cycle when *m* is an equal value for all cycles. Accordingly, if *m* is the same in every dataset, the parameters associated with the sagittal and transverse planes, *S* and *T*, respectively, in Figure 1 are averaged in the final step of the analysis protocol to generate a standard gait pattern.

**Figure 6:**
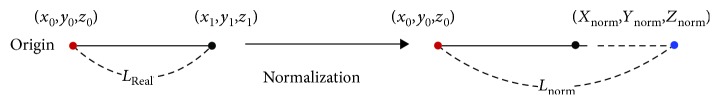
Normalization of body segment length.

The dataset also needed to be normalized in space to standardize the trajectories of the joints because the length of each body segment is different from the other. Hence, the positional trajectories of the joints were reconstructed by obtaining the equivalent lengths of each body segment.  Figure 6 expresses the method for normalization of the body segment length.

A real segment length, *L*_Real_, from reference point *P*_0_ = (*x*_0_, *y*_0_, *z*_0_) to the other point *P*_1_ = (*x*_1_, *y*_1_, *z*_1_) was rearranged to a new point *P*_(norm)_ = (*x*_norm_, *y*_norm_, *z*_norm_) with the desired length *L*_(norm)_. We decided *L*_(norm)_ to be the average value of the length of the lower leg and thigh in Table 1. The relation between normalized point *P*_(norm)_, the reference point *P*_0_, and the new point *P*_1_ is shown in (1) and the normalized dataset *D*_(norm)_ was computed through the equation given in [38].

(1) $$\begin{array}{c} P_{\left(\mathrm{norm}\right)}=P_{0}-\frac{L_{\mathrm{norm}}}{L_{\text{Real}}}\left(P_{0}-P_{1}\right). \end{array}$$

### 2.3. Parameters for Motion Analysis

The hip, knee, and ankle joints were mainly characterized by large ranges of motion (ROMs) in the sagittal plane rather than in the coronal or transverse mobility [9, 18–20]. Despite the small actions on the transverse plane, it is important that hip movement can contribute to the advancement of muscle strength and effective balance training [39]. Thus, the parameters for analysis of motion on the transverse plane, in particular the hip joint, as well as that on the sagittal plane were examined. Four parameters were considered in this study: joint flexion/extension angle and positional trajectory (on the sagittal plane), tendency of hip translation, and hip rotation (on the transverse plane). These were determined by the relevant positions either to the sagittal plane [*Y*_(norm)_ *Z*_(norm)_] or to the transverse plane [*X*_(norm)_ *Z*_(norm)_].

**Figure 7:**
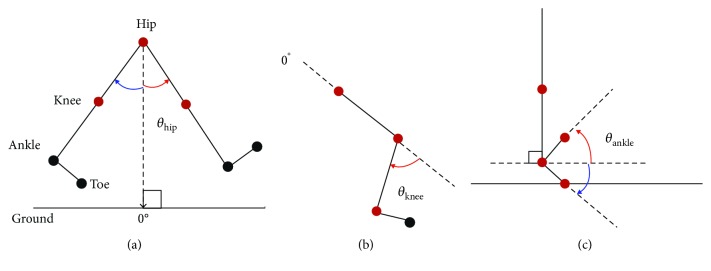
Definition of joint angles *S*_(ang)_: (a) flexion/extension of hip joint *θ*_hip_, (b) flexion/extension of the knee joint *θ*_knee_, and (c) dorsi-/plantar-flexion of ankle joint *θ*_ankle_. The red points indicate joints, and the red/blue arrows denote the positive/negative sign of angular direction.

The first parameter was angular trajectory *S*_ang_ = [*θ*_hip_, *θ*_knee_, *θ*_ankle_], which signifies the trend of the hip, knee, and ankle during a stride on the stair. The angular trajectory was obtained from the first law of cosines. The directions indicated in Figure 7 and the following conditions defined these angles and their signs (positive/negative):

- If the hip joint poses on hip flexion, *θ*_hip_ > 0
- If the knee joint poses on knee flexion, *θ*_knee_ > 0
- If the ankle joint poses on dorsiflexion, *θ*_ankle_ > 0

The joints of the robot should be designed to move in a closed-loop pattern to generate a repetitive gait motion in the fixed system even if the resulting data from the experiment is an open curve. For this reason, the trajectories of the joints, as secondary parameters, were replaced with relative positions from a point for stair-gait patterns during a circular walk. The reference point was set as the hip marker position. In other words, the position of the hip is considered as (0, 0) and the positions of the knee and ankle, which were secondary parameters, moved relatively to the reference point.

**Figure 8:**
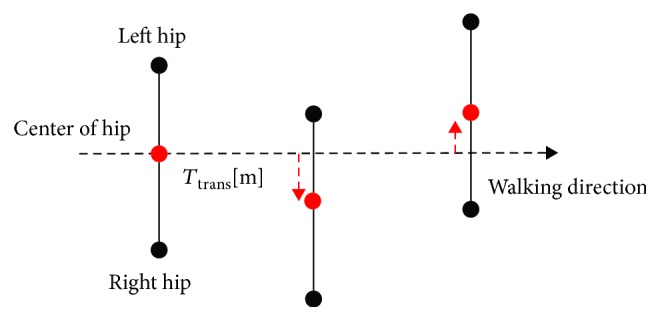
Definition of mediolateral movement, *T*_trans_.

In general, most existing robotic locomotion rehabilitation systems address the kinematics on the sagittal plane because the lower limb is akin to working predominantly for flexion/extension during locomotion. Such a movement constrained to only one anatomical plane can prevent meaningful training for more effective therapeutic impact. The hip joint, especially, has distinct movement on the transverse plane owing to weight bearing or weight shifting during walking. Among the features of relevance to the robotic gait-training system [39], the hip translational movement, *T*_trans_, in the mediolateral direction is considered as the third parameter.  Figure 8 shows the method used to calculate the variation of hip movement on the transverse plane. The length between the left and right hip markers is considered a constant because it is an intrinsic value as the length of a body segment. The variation of mediolateral hip movement can be measured in terms of displacement of the center of the hip segment.

Although the participants performed stair walking in the same coordinates and location, the planes on which their trajectories were described were not exactly coincident. In other words, the walking directions for all the data sets were different. Therefore, the data sets were manipulated so that they were in the same sagittal plane using the rotational displacement formula [40]. Thus, the right and left hip markers made a line, and the center point on the line drew a curve along weight shift. Then, trends of positional variation of the center point between the hip joints in the same walking direction could be determined.

**Figure 9:**
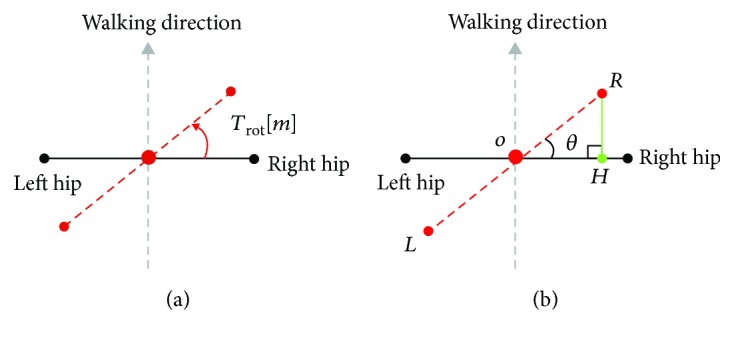
Definition of hip rotation angle *T*_rot_: *T*_rot_ in (a) equals the included angle *θ* of the right triangle *∆ROH* in (b).

The last parameter for the motion analysis is the angular displacement associated with the hip rotation during gait.  Figure 9 indicates the methods for calculating the variation of hip rotation on the transverse plane. The hip rotation, *T*_rot_, was defined as the angle between the line perpendicular to the walking direction and the line of hip markers. The rotation angle was determined by making a right triangle and finding the included angle with the inverse tangent function as shown Figure 9(b). The parameter was defined as a positive value where the right hip marker was placed in front of the left hip marker.

The result of the data processing such as normalization and interpolation makes trajectories for a gait cycle, but it might not be appropriate to be applied to a fixed type rehabilitation robot. If values in the beginning and end points of the trajectories are different, they make a discontinuity when the robot is working because the robot needs a cyclic gait pattern. Therefore, the points of the beginning and the end points on all results should match to make a cyclic pattern. To resolve this problem, the obtained datasets were processed by the cubic spline method using the points corresponding to the first 5% (0 to 5%) and the last 5% (96 to 100%) of the stride cycle.

## 3. Results

### 3.1. Angular and Positional Trajectories of Joints on the Sagittal Plane

**Figure 10:**
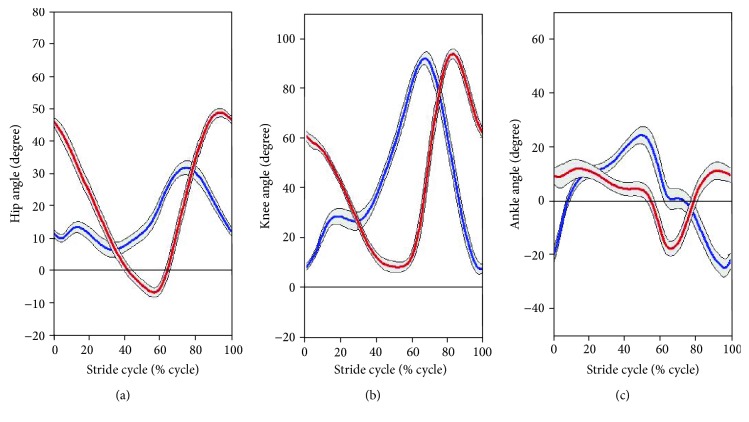
Mean angles of the (a) hip, (b) knee, and (c) ankle joint: the blue lines indicate the variation of the joint angle during stair ascent, and the red lines indicate the variation of the joint angle during stair descent.

As mentioned in the previous section, we calculated two parameters of joint angles and trajectories on the sagittal plane to analyze stair-walk motion.  Figure 10 shows variations in the hip, knee, and ankle joint angles during stair ascent (red line) and stair descent (blue line), and their standard deviations are given by the gray areas. In this study, the average ROMs for the subjects' hip joints in extension/flexion during a stair ascent and descent cycle were (−6.75°, 48.69°) and (6.41°, 31.67°), respectively. The average ROM of the knee joints in extension/flexion was (8.20°, 93.78°) during stair ascent and (7.38°, 91.93°) during stair descent. Additionally, the average ROMs of ankle joints in plantar-/dorsiflexion were (−17.78°, 11.75°) and (−24.89°, 24.18°) during stair ascent and descent, respectively.

**Figure 11:**
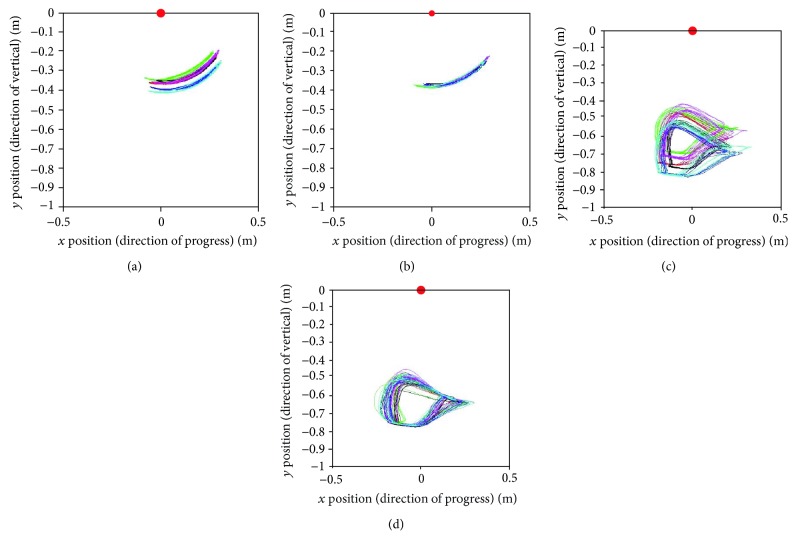
Relative trajectories from the hip joint during stair ascent: (a) knee trajectories and (c) ankle trajectories of each subject. (b and d) Knee and ankle trajectories are shown as a result of normalization for the lengths of the body segments.

**Figure 12:**
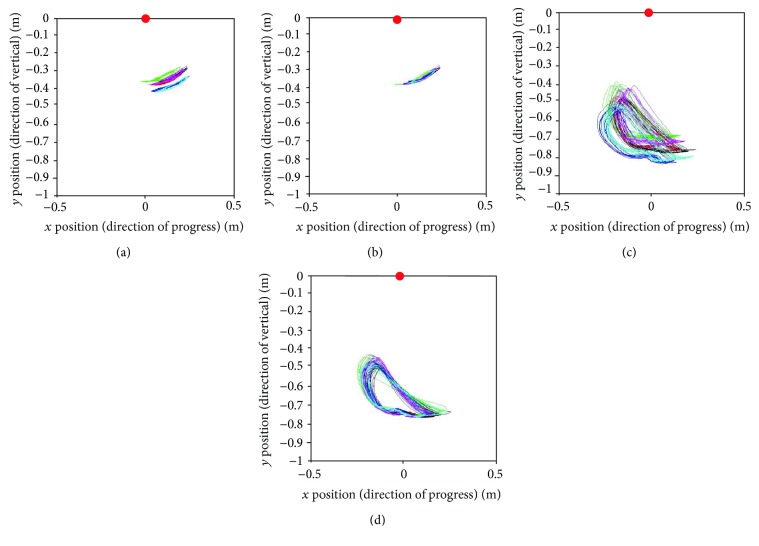
Relative trajectories from the hip joint during stair descent: (a) knee trajectories and (c) ankle trajectories of each subject. (b and d) Knee and ankle trajectories are shown as a result of normalization for the lengths of the body segments.

Figures 11 and 12 present the relative trajectories of the knee and ankle joints for the hip joint on the sagittal plane during stair ascent and descent, respectively. The different colors of trajectories in Figures 11 and 12 present different subjects. To reduce the individual variation in the lengths of the body segments, the data were normalized with the algorithm described in Section 2.2. The red points on these figures represent the hip marker at the reference point (0, 0).

**Figure 13:**
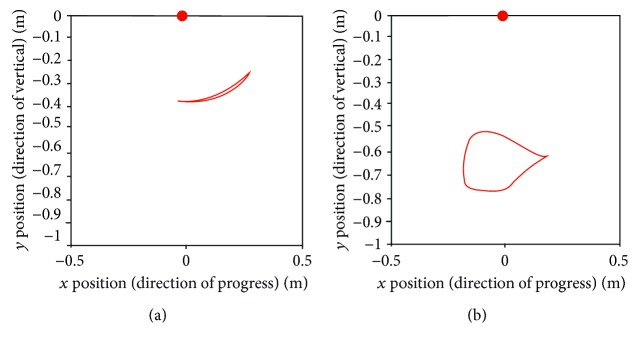
Standard trajectories of the (a) knee and (b) ankle during stair ascent.

**Figure 14:**
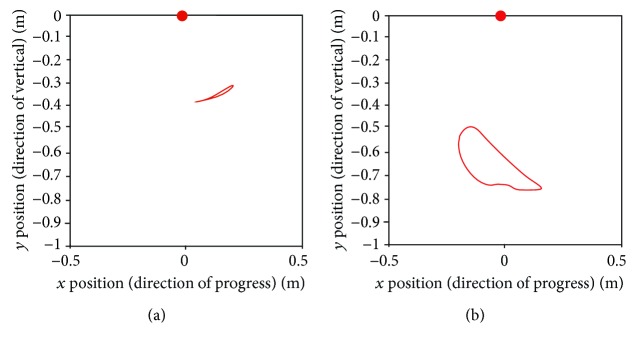
Standard trajectories of the (a) knee and (b) ankle during stair descent.

After normalization, we attempted to find the standard trajectories of the knee and ankle. As shown in Figures 13 and 14, the averaged trajectories of the normalized datasets, the red lines, are considered the standard trajectories in this experiment.

### 3.2. Hip Movement on the Transverse Plane

**Figure 15:**
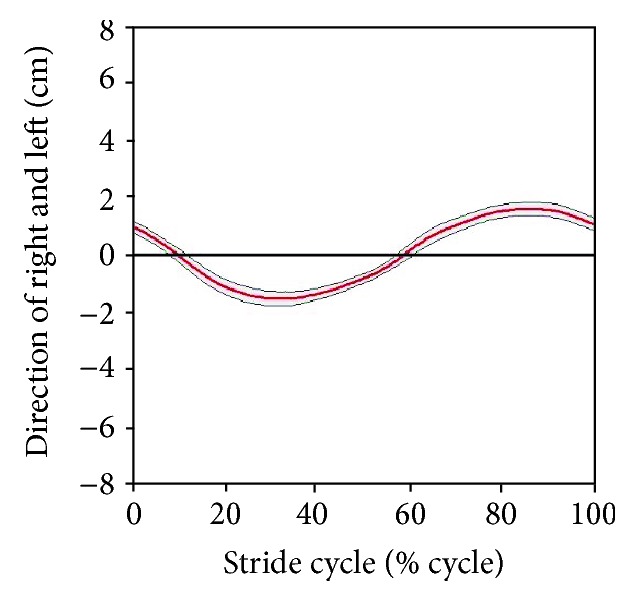
Variation of hip translation during stair ascent.

**Figure 16:**
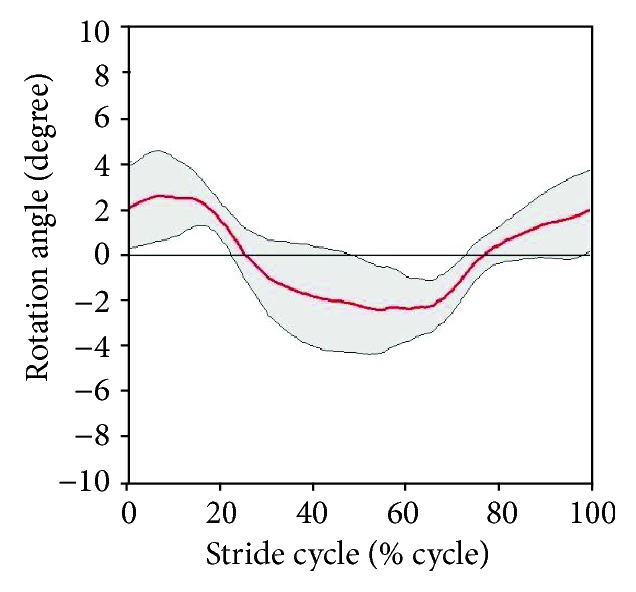
Variation of hip rotation during stair ascent.

Figures 15 and 16 present the variation in hip translation and rotation, respectively, during a stair-ascent cycle. The translation/rotation is indicated by the red line. The standard deviation is indicated by gray lines. When ascending a stair, the averaged ROMs on the transverse plane were within ±1.57 cm for translation and ±2.52° for rotational movement.

**Figure 17:**
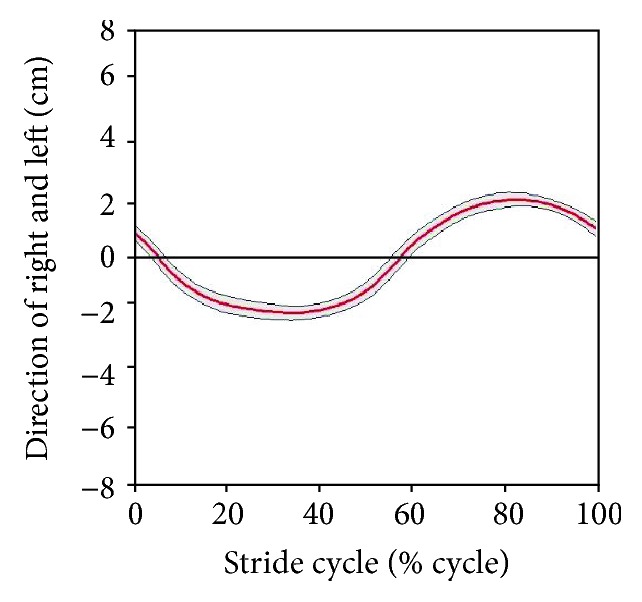
Variation of hip translation during stair descent.

**Figure 18:**
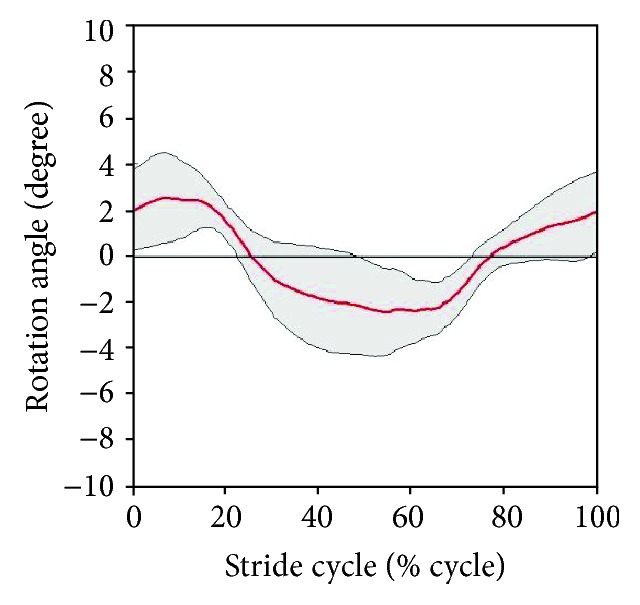
Variation of hip rotation during stair descent.

As with Figures 15 and 16, Figures 17 and 18 indicate trends in the hip movement for a stair-gait cycle. The range of translation movement was estimated to be within ±2.00 cm, and hip rotation was estimated to be within ±2.70°.  Table 2 shows the maximum range in which subjects actually moved in the experiment.

Table 2 shows the minimum and maximum values of data, which consist of the resampled 120 datasets from the experiment. The values in Table 2 cover the range of all subjects' motion.

Because the gait cycle was divided into 200 phases to derive the pattern of stair walking, standard deviation values were different for each point in Figure 10 and Figures 15–18. Thus, the principal estimation of standard deviations for each result for each motion is summarized in Tables 3 and 4.  Table 3 shows the maximal and minimal values of standard deviations for each subject.  Table 4 presents the principal estimations of standard deviation on each result in Figure 10 and Figures 13–18.

### 3.3. Application of Derived Pattern to the Robotic System

**Figure 19:**
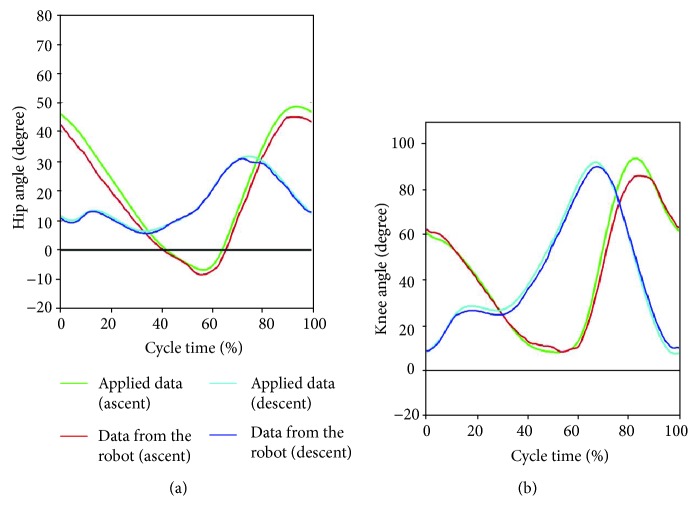
Comparison of the angular trajectories on (a) hip joint and (b) knee joint between robot movement and experimental data.

If the trajectory is compared with the joint displacement data of a robotic training system served by itself, it can ascertain whether the system properly works within a normal ROM, e.g., the height of a leg lift. Actual angular trajectories performed by the robotic system designed for stair walking during stair ascent and descent are displayed in Figure 19. The trajectories generally follow the gait pattern obtained from this study (green and light blue line) even though there is some delay or errors—average errors within ±8% were calculated.

## 4. Discussion

In this study, we attempted to create patterns of stair walking for application to a robotic lower-limb rehabilitation system. A subject's legs moved in a cyclical pattern during stair negotiation. The movement of the lower limb primarily appears as a flexion/extension of each joint [20]. Therefore, initially, variations in the joint angles of the hips, knees, and ankles were extracted on the anatomical sagittal plane such that the robotic exoskeleton of the gait-training system can work with the most basic gait pattern. The calculated angular variations of the hips, knees, and ankles, as shown in Figure 10, were used to establish the basic pattern in stair ascent and stair descent.

As shown in Table 1, the subjects had different stride lengths and leg lengths in the stair-walk experiment. Therefore, we normalized the lengths of body segments before calculating the knee and ankle trajectories relative to the hip. As shown in Figures 11 and 12, it was easy to find the trend of the normalized knee and ankle joint trajectories. Additionally, the normalization is supposed to establish criteria for the gait pattern to drive a robotic gait trainer after standardization of the relative trajectories. Figures 13 and 14 show the desired tracks of the knee and ankle joints for a robotic system mimicking the experimental pattern in Figure 10.

In addition to the analysis on the sagittal plane, we tried to examine the hip joint on the transverse plane. The medial-lateral movements of the hip during stair walking seemed to be similar among the subjects, as shown in Figures 15 and 17. However, the variation in hip rotation angles had large standard deviations, as shown in Figures 16 and 18. This is due to differences in the gait patterns of each individual, such as step length, body segment length, gender, and other anatomical factors. Its effectiveness should be investigated by a clinical test, which, however, is beyond the scope of this work.

The exoskeleton of the robotic system was designed based on the results shown in Table 2, and it could move within a range that covered all subjects. As shown in Tables 3 and 4, standard deviations on the sagittal plane in Table 3 are larger than those in Table 4, and the results on the transverse plane in Table 4 are larger than those in Table 3. It means that the standard patterns on the sagittal plane reflected the general trend of stair walk, and the variation within an individual on the transverse plane is larger than among subjects. Therefore, each joint of the exoskeleton was controlled by a standard pattern in Figure 10 for reflecting general patterns on the robotic system. On the other hand, hip movements on the transverse plane were controlled within ranges of standard deviations depending on the individual difference as shown in Figures 15–18.

As compared to the motion of a robot with the derived standard pattern shown in Figure 19, the trend of the motion between the applied data and that measured from the robot is almost similar, but some inevitable errors occurred. These errors are considered to be due to variations in the measuring or control method in the robot.

## 5. Conclusions

The present study has shown the process of analysis and the method for acquiring the motion patterns of lower limbs during stair walking. The ROMs determined through this study covered the clinically known ROMs in accordance with each gait phase [20, 25, 41, 42]. Consequently, we concluded that our experimental results indicate normal stair-gait patterns for the hip, knee, and ankle on the sagittal plane. However, there are several features that should be considered when analyzing hip rotation because it tends to be more influenced by diverse individual walking habits or body type. Therefore, we need to experiment further with algorithms that consider various factors when determining the normal gait pattern of a rotated hip during stair walking. Moreover, further research is required on the application of the obtained data to a robot to ascertain whether natural stair-walk training is possible after an additional study has been conducted on hip rotation.

## Figures and Tables

**Table 1:** Information about each subject.

Subject no.	Gender	Length of the thigh (cm)	Length of the lower leg (cm)
Sub 1	Male	36.67	38.09
Sub 2	Female	34.41	33.85
Sub 3	Male	40.04	41.69
Sub 4	Male	36.38	40.79
Sub 5	Female	36.19	35.08
Sub 6	Male	40.81	39.90

Mean value of the length (standard deviation)		37.42 (2.47)	38.23 (3.18)

**Table 2:** ROM on all subjects applying to the motion of the robotic system.

	Stair ascent		Stair descent	
	Min.	Max.	Min.	Max.
Hip angle	-14.87°	56.10°	-4.62°	40.18°
Knee angle	0.051°	104.11°	0.0048°	104.14°
Ankle angle	-36.93°	24.13°	-37.87°	35.87°
Hip translation	-2.68 cm	2.68 cm	-3.17 cm	3.17 cm
Hip rotation	-16.71°	16.66°	-10.60°	10.29°

**Table 3:** Principal standard deviation within each subject.

	Sub 1		Sub 2		Sub 3		Sub 4		Sub 5		Sub 6	
	Min.	Max.	Min.	Max.	Min.	Max.	Min.	Max.	Min.	Max.	Min.	Max.
Stair ascent												
Hip angle	1.16	5.02	1.62	8.12	0.64	6.89	1.17	6.89	1.17	6.50	1.04	5.60
Knee angle	1.18	11.83	0.86	16.41	1.12	15.85	0.89	10.16	1.44	7.69	0.79	4.99
Ankle angle	0.51	7.77	1.10	8.66	1.30	8.89	1.35	8.54	0.61	7.54	0.36	4.17
Hip trans.	0.15	0.39	0.10	0.49	0.15	0.46	0.14	0.68	0.21	0.69	0.17	0.32
Hip rotation	1.31	6.52	1.09	4.47	0.14	2.00	0.27	2.70	0.02	2.50	0.32	2.72

Stair descent												
Hip angle	0.63	3.26	0.91	5.29	0.68	4.92	1.19	5.09	1.26	3.45	0.53	2.79
Knee angle	0.81	8.03	1.59	10.42	1.05	15.27	1.51	11.79	0.93	5.84	0.61	6.81
Ankle angle	1.09	6.27	1.15	6.78	1.20	8.69	2.49	11.49	0.44	6.78	0.33	5.57
Hip trans.	0.21	0.28	0.13	0.87	0.29	0.91	0.16	0.90	0.07	0.68	0.14	0.61
Hip rotation	0.17	1.35	0.51	1.77	0.41	1.70	0.73	3.28	0.06	3.00	0.11	1.25

**Table 4:** Principal standard deviation of all subjects.

	Stair ascent		Stair descent	
	Min.	Max.	Min.	Max.
Hip angle	2.12	6.28	2.30	4.86
Knee angle	2.96	12.22	2.55	11.18
Ankle angle	4.57	8.70	3.73	11.10
Hip translation	0.32	0.53	0.38	0.69
Hip rotation	1.47	4.42	1.80	3.17

## Data Availability

The kinematic data used to support the findings of this study are available from the corresponding author upon request.
